# Bioinformatics Mining and Modeling Methods for the Identification of Disease Mechanisms in Neurodegenerative Disorders

**DOI:** 10.3390/ijms161226148

**Published:** 2015-12-07

**Authors:** Martin Hofmann-Apitius, Gordon Ball, Stephan Gebel, Shweta Bagewadi, Bernard de Bono, Reinhard Schneider, Matt Page, Alpha Tom Kodamullil, Erfan Younesi, Christian Ebeling, Jesper Tegnér, Luc Canard

**Affiliations:** 1 Department of Bioinformatics, Fraunhofer Institute for Algorithms and Scientific Computing (SCAI), Institutszentrum Birlinghoven, Sankt Augustin D-53754, Germany; shweta.bagewadi@scai.fraunhofer.de (S.B.); erfan.younesi@scai.fraunhofer.de (E.Y.); christian.ebeling@scai.fraunhofer.de (C.E.); 2 Rheinische Friedrich-Wilhelms-Universitaet Bonn, University of Bonn, Bonn 53113, Germany; alpha.tom.kodamullil@scai.fraunhofer.de; 3 Unit of Computational Medicine, Center for Molecular Medicine, Department of Medicine, and Unit of Clinical Epidemiology, Karolinska University Hospital, Stockholm SE-171 77, Sweden; gordon.ball@ki.se (G.B.); jesper.tegner@ki.se (J.T.); 4 Science for Life Laboratories, Karolinska Institutet, Stockholm SE-171 77, Sweden; 5 Luxembourg Centre for Systems Biomedicine, University of Luxembourg, 7, avenue des Hauts-Fourneaux, Esch-sur-Alzette L-4362, Luxembourg; stephan.gebel@uni.lu (S.G.); reinhard.schneider@uni.lu (R.S.); 6 Institute of Health Informatics, University College London, London NW1 2DA, UK; b.bono@ucl.ac.uk; 7 Auckland Bioengineering Institute, University of Auckland, Symmonds Street, Auckland 1142, New Zealand; 8 Translational Bioinformatics, UCB Pharma, 216 Bath Rd, Slough SL1 3WE, UK; matthew.page@ucb.com; 9 Translational Science Unit, SANOFI Recherche & Développement, 1 Avenue Pierre Brossolette, Chilly-Mazarin Cedex 91385, France; luc.canard@sanofi.com

**Keywords:** mechanism-identification, bioinformatics, genetics, graphical models, knowledge-based modeling, multiscale, neurodegeneration, data integration, disease models

## Abstract

Since the decoding of the Human Genome, techniques from bioinformatics, statistics, and machine learning have been instrumental in uncovering patterns in increasing amounts and types of different data produced by technical profiling technologies applied to clinical samples, animal models, and cellular systems. Yet, progress on unravelling biological mechanisms, causally driving diseases, has been limited, in part due to the inherent complexity of biological systems. Whereas we have witnessed progress in the areas of cancer, cardiovascular and metabolic diseases, the area of neurodegenerative diseases has proved to be very challenging. This is in part because the aetiology of neurodegenerative diseases such as Alzheimer´s disease or Parkinson´s disease is unknown, rendering it very difficult to discern early causal events. Here we describe a panel of bioinformatics and modeling approaches that have recently been developed to identify candidate mechanisms of neurodegenerative diseases based on publicly available data and knowledge. We identify two complementary strategies—data mining techniques using genetic data as a starting point to be further enriched using other data-types, or alternatively to encode prior knowledge about disease mechanisms in a model based framework supporting reasoning and enrichment analysis. Our review illustrates the challenges entailed in integrating heterogeneous, multiscale and multimodal information in the area of neurology in general and neurodegeneration in particular. We conclude, that progress would be accelerated by increasing efforts on performing systematic collection of multiple data-types over time from each individual suffering from neurodegenerative disease. The work presented here has been driven by project AETIONOMY; a project funded in the course of the Innovative Medicines Initiative (IMI); which is a public-private partnership of the European Federation of Pharmaceutical Industry Associations (EFPIA) and the European Commission (EC).

## 1. Introduction

Integrating disease relevant data for mining and modeling approaches has emerged as a major research area ranging from biology, translational medicine, and pharmaceutical industry. Rationales include finding new biomarkers for diagnostics, prognosis or response to therapy and potentially increased understanding of disease, thereby paving the way for developing new therapeutics [1]. Yet, since diseases are as a rule multi-factorial, progress on unraveling causally relevant biological mechanisms has been even more challenging compared to identifying biomarkers. Recently, there have been significant advances made in the areas of cancer, cardiovascular and metabolic diseases. For example, collecting data such as the cancer genome atlas [2] has set the stage for a large number of integrative studies finding biomarkers and processes associated with progression of cancer [3,4,5,6]. Schadt and colleagues have advanced the precision in identifying disease relevant genomic mechanisms in cardiovascular and metabolic diseases using Bayesian networks [7,8]. Overall, it is not yet clear how to integrate data in specific cases since it does depend on the available data and questions that one wants to answer. However, to advance beyond putative biomarkers towards a mechanistic understanding it is desirable to use a model-based approach which is challenging due to the biological complexity and sparseness of data [9].

These challenges become abundantly clear in the case of neurodegenerative diseases in part because they are “idiopathic” diseases, which means, that their aetiology is not really understood. The fact that neurodegenerative diseases—at least in their sporadic (non-familial) form—are “slow” diseases that show incubation (latency) times of more than 20 years [10], makes research on the aetiology of these diseases such a challenging undertaking. Histopathology studies, epidemiological evidences, genetic analysis and clinical observations suggest that neurodegenerative diseases have several mechanisms [11]; and that there is not a single “cause-effect” relationship that would explain the full spectrum of pathological changes that can be observed in the patient. Although genetic analysis of familial forms of Alzheimer´s disease (AD) and Parkinson´s disease (PD) has shed some light on the molecular mechanisms that are likely to contribute to the aetiology of the disease [12,13], these mechanisms cannot account for the spectrum of pathologies associated with sporadic forms of neurodegenerative diseases. In fact, the debates about some of the putative mechanisms that may be involved in early dysregulation events (e.g., the role of sterile inflammation in the brain [14]; or the debate on prion-like spreading of the disease [15]) indicate the complexity and diversity of routes that lead to the dramatic changes that we observe in later stages of dementias.

It is therefore a widely accepted notion in the scientific community, that neurodegenerative diseases are complex diseases presumably involving a multitude of mechanisms that execute their function at different levels. These may span from dysregulation at the molecular level [16] via perturbed cell-cell interactions (including neuro-inflammatory processes) [17] to pathophysiological changes at organ level, e.g., the blood vessel system [18]. Any attempt at understanding neurodegeneration at a mechanistic level therefore has to be inherently multilevel. With respect to the detection technologies used to generate the data specific for each level, the modeling and mining techniques need to be able to deal with multiple scales (e.g., nanosecond (time) and atomic (space) scale to years (time) and the entire organ physiology (space) [19]). Variables (readouts) on these different scales are measured using different detection modes, ranging from imaging via biomarker measurements to cognitive testing. Hence, methods of data-integration have the potential to significantly advance the state-of-the-art and set the stage for a more unbiased search of biomarkers and processes associated with neurodegenerative diseases.

Yet, mechanistic understanding of disease aetiology requires going way beyond what is known as biomarker identification and biomarker qualification. Per definition, a biomarker is (*sic*) “a characteristic that is objectively measured and evaluated as an indicator of normal biological processes, pathogenic processes or pharmacological responses to a therapeutic intervention” [20]. Biomarkers correlate with a state and their presence or absence says something about the state, a cell, tissue or organ (or an entire organism) is at a given time point. However, a biomarker is neither necessarily part of a mechanism, nor does it naturally represent a mechanism. The type of relationship that exists between a biomarker and the biological process it reflects is from a logical point of view “<*correlates_with*>”. Biomarkers may therefore be useful to define patient subgroups according to their discriminating potential (prognostic biomarkers). If a biomarker is strongly associated with a target for intervention, the measurement of a biomarker does allow for a prediction of treatment responses (predictive biomarker). However, even in the case of predictive biomarkers, the measured entity does not necessarily need to be causally involved in a disease mechanism. From a machine learning perspective, there exist features that are statistically predictive without being causally involved [21]. For example, blog entries were shown to be predictive of spread of influenza despite not being causally related to the mechanisms of influenza [22].

In contrast, a biological disease mechanism, is indispensably linked to a causal relationship. A “chain of causes and effects” forms a pathophysiological context, where minor dysregulation of molecular events may aggregate at a network level and lead to a pathological deviation from the normal state [23]. If the effect sizes of these dysregulatory events are high (e.g., in monogenic diseases), the “chain of causes and effects” is most likely short and direct, as a consequence, the link between the event and the disease phenotype is often less complicated. In complex diseases, however, effect sizes are as a rule small and multiple events contribute to a dysregulation, that may take years to manifest at the clinical phenotype level. In this case, dysregulation at the network level may involve different types of entities; so-called “intermediate phenotypes” (molecules, protein complexes, biological processes) [24].

Different types of predicates are used to model cause-and-effect relationships; in general, all sorts of “regulation” relationships (“activation”; “induction”; “increase”; and “inhibition”; “suppression” and “decrease”) are indicative for cause-and-effect relationships. At the graph level, these relationships are represented as directed graphs. We should be aware, that the statement “causes_no_change” is also a causal statement, and as such constitutes a (lack of) relationship.

In complex diseases such as neurodegenerative diseases, we are most likely dealing with several mechanisms that contribute to the aetiology of the disease. Such multitude of cause-and-effect relationships, can be represented and captured in the language of directed graphs [25], thus providing a framework in which their putative crosstalk and mutual influence can be investigated. Further complication of the situation may stem from modifier functions (e.g., Single Nucleotide Polymorphisms (SNPs) or epigenetic variants that have very small effect sizes) that have an influence on onset and progression of disease without dramatically changing the essential mechanistic aetiology of the disease. An individual patient is therefore likely to represent a complex mixture of causal and correlative relationships between entities (“factors”) that display different impact and that exist at different levels (e.g., ranging from genetic variation that contributes to overall risk [26], via behavioral factors such as smoking and physical exercise [27] to risk that is associated with viral infections [28] or the gut microbiome [29] indicated by epidemiological and genetics evidences).

As outlined above, disease mechanisms are likely to involve factors from different levels of biology. This makes it unlikely that measurements at only one level (e.g., only mRNA expression or only proteomics measurements of certain a β fragments) can adequately capture the entire spectrum of mechanisms. This becomes evident, when considering “higher order biological processes” that have been hypothesized to play a role in neurodegeneration such as “autophagy” or “mitochondrial stress”. A measurement of mRNA expression would therefore only provide an incomplete picture about autophagy and its involvement in neurodegenerative processes; and proteomics measurements would hardly reveal the complex biological (dys-)regulation associated with “mitochondrial stress”. It is noteworthy at this point to underline that analysis of differential gene expression would not have identified the amyloid cascade, one of the key processes associated with Alzheimer´s disease, as a candidate mechanism, as the genes involved in the amyloid cascade do not show a strong correlation with the pathological state in gene expression studies [30].

As a consequence, we should envision mechanisms as causal relationship graphs that involve all levels of biology, from genetic variation and epigenomics patterns via proteolytic processing and cellular physiology to tissue and organ (patho-) physiology.

Below, we review two different classes of promising bioinformatics approaches targeting causal disease driving mechanisms in the context of neurodegenerative diseases. One strategy is to benefit from advances in genetics and in particular how to utilize current SNP databases to widen the search for putative disease-causing mechanisms. The rationale is that genetics constitutes one of the most robust and high-quality data-sets in our possession. At the other end of the spectrum, there exists a mechanistic model-driven approach, fuelled by prior knowledge from experts and text mining strategies, which collects and organizes putative mechanisms in a formal framework. Here we review different kinds of approaches towards candidate mechanism identification; some of which are based on formal frameworks such as Open Biological Expression Language (openBEL), Resource Description Framework (RDF), and physiology representations in an anatomy context. In addition, we demonstrate how this diverse set of mechanism-identification approaches has been integrated in the AETIONOMY Knowledge Base, providing unprecedented capabilities of candidate mechanism identification in the challenging area of neurodegenerative disease biology.

## 2. Bioinformatics Methods for the Identification of Disease Mechanisms

### 2.1. Availability of Data for Mechanism Identification

Clearly, if we would have detailed data from every patient, ranging from molecules to lifestyle during an extended time-course prior onset and during progression of disease, then we would be in a powerful situation to disentangle causal mechanisms from correlative phenomena, as well as being able to stratify different routes to disease for different groups of patients. However, thus far, attempts at identifying pathophysiology mechanisms in neurodegenerative diseases have largely been building on measurements at a single level (with a single readout based on a single measurement technology) and in the best case in more than a single time-point. The accessibility and availability of a body fluid or a tissue sample determined to a large extent at what time and with what type of technology a certain measurement is done. This brings to the field a strong bias, e.g., towards using certain body fluids (e.g., cerebrospinal fluid (CSF) for proteomic analysis and miRNA expression studies) and certain time points or as often is a rule, a single time-point (e.g., post-mortem brains). Importantly, in larger studies such as Alzheimer’s Disease Neuroimaging Initiative (ADNI) [31], the scientific community has recently begun to study clinical data (including assessment of cognitive functions), neuro-imaging and genetics simultaneously. Ongoing studies now combine advanced imaging technologies that allow for detection of pathophysiology factors (e.g., PET amyloid), cognitive function assessment and measurements of individual genetic variance (predominantly SNP measurements) [32,33]. These “multi-modal” data sets will play an essential role in integrative modeling and mining approaches aimed at mechanism-identification in the future. Here we remark that similar efforts have been the rule in the cancer community and the creation of the cancer genome atlas incorporating several different layers of data-types [34] has spearheaded a rapid progress in discovering both biomarkers and putative causal mechanisms as well has spurred the development of new methods for integrative data-analysis. Hence, to set the stage for a similar development in the areas of neurodegenerative disease, emerging and current efforts need to be further supported and extended not only with regard to multi-modality of data but also to enable monitoring of patients over time, while carefully collecting clinical parameters. Here we conclude and advise that upcoming studies should be carefully designed with these considerations in mind if we are to succeed in identifying causal mechanisms thus advancing beyond biomarker discovery.

### 2.2. The Gap between Genetics and Mechanisms of Disease

It is clear that candidate gene association studies and genome-wide association studies (GWAS) provide statistically robust associations between genetic variants and clinical phenotypes. Supported by the strong effect that familial mutations in disease-associated genes exhibits (e.g., Presenilin mutations, *etc.*, in AD [10] and Parkin mutations in PD [35]), numerous studies have attempted at identifying additional allelic variants and mutations that may account for the genetic predisposition in sporadic disease [36]. The design of genetic studies in neurodegenerative diseases is following a well-established schema: a healthy control cohort and a “disease cohort” are being selected based on inclusion and exclusion criteria defined in the course of study design. Both cohorts will be characterized by clinical variables (e.g., neuro-imaging, cognitive testing; cognitive decline over time) and the panel of clinical variables is correlated with SNP data (typically, a “genome-wide” SNP detection assay measure up to one million SNPs simultaneously).

SNPs are being associated with the clinical phenotype using statistical approaches (reviewed in [37]); the sensitivity of these analyses depends much on the cohort size and typically only the most significant SNPs are being investigated in detail. Further filtering (e.g., by selecting only those SNPs that are in genomic regions that display “open chromatin”; usually making use of ENCODE data [38]) aims at accumulating supportive evidence that a statistically significant SNP is truly associated with disease. Often, “disease-associated loci” are being identified by GWAS analysis; only rarely does the analysis directly lead to a clear association of a SNP with a gene (as most of the SNPs are intergenic SNPs).

Meta-analysis of GWAS data has the potential to unravel new disease-associated loci [39] by effectively increasing the statistical power of this sort of analysis.

Common to all statistical approaches is that they aim at associating genetic variance information with a disease cohort (characterized by some disease associated clinical readouts) and generate “candidate genes” or “candidate loci” for disease susceptibility. Candidate genes need to be associated with biological processes and pathways in order to establish a biologically meaningful interpretation of the role of SNPs in the disease. This step in the analysis of SNP data is the most challenging one, for several reasons:

- The functional impact of a SNP cannot easily be assessed for intergenic SNPs [40]. As a consequence, reasoning over the mechanistic consequences of intergenic SNPs is a non-trivial undertaking [41].
- The purely statistical approach of associating SNPs with disease (or better: syndromes, as they are likely to have multiple aetiologies) tends to ignore SNPs that play a role only in a small subgroup of patients. In complex diseases and in particular in neurodegenerative diseases, we have reasons to assume that such subgroups of patients exist [42]. If the dysregulation of several pathways together constitutes the disease phenotype, we may deal with several “rare” and “low effect size” SNPs that act in a cooperative fashion and jointly contribute to the aetiology of the disease. Hence, a population based statistical approach can therefore readily be expected to be associated with a fair amount of false negatives, *i.e*., SNPs escaping the analysis.
- Any functional impact assessment of intergenic SNPs, using an enrichment approach, requires a substantial number of examples of intergenic SNPs that have already been characterized. Such a “knowledge base” of intergenic SNPs characterized at the mechanistic level is currently not available, and one strategy to mitigate this gap is to develop algorithms predicting the functional impact of intergenic SNPs and other genetic variants [43].

Although we do acknowledge the important role that genetics analysis in general and GWAS in particular has played in the process of identifying candidate genes and candidate loci that may play a role in disease, this sort of incomplete information does not in itself necessarily represent “disease mechanisms”. Candidate genes need to be characterized by a functional context, either through association with “pathways” or by other knowledge structures that contain cause-and-effect relationships. Therefore, the analysis of genetic variance may play an important role in mechanism-identification, but it is unlikely to deliver the insights we need to establish cause-effect relationships that are responsible for the aetiology of a disease.

#### 2.2.1. Strategies for Advancing beyond Individual SNPs towards Disease Mechanisms

There is huge interest in the research community to elucidate the underpinnings between genetic and phenotypic variations as evidenced from numerous studies across different diseases. As nicely reviewed recently [44] these methods utilize several omics data-types such as transcriptomics, epigenomics, and proteomics in order to bridge the gap between genetics and phenotypes. Using several data-types either at once or in a sequential analysis may thus yield biomarkers and potentially suggest tissues or molecular pathways associated with the disease. For example, if there are several data-types such as genetics, transcriptomics, and clinical readouts available for every individual, then a Bayesian scheme is very powerful approach since it integrates all these data-types at once [7]. However, in the case of neurodegenerative disease we are instead faced with sparse incomplete and heterogeneous data and a given individual is thus represented by a thin or sparse data-set. This makes a sequential approach more useful where different kinds of data from different sources are collected and fused. Below we illustrate this approach in the context of Alzheimer´s disease where the concept is to use SNPs as a starting point for collecting additional data-types in the molecular “vicinity” of those SNPs. The idea is to capture biological processes, which potentially are involved in AD.

Hence, using reliable population derived SNP´s as an established basis, we incorporated several data-points from other public data types based on their vicinity to those population SNP´s, thereby augmenting the identification of disease relevant molecular processes. These other data-types and procedures include the identification of the associated Linkage Disequilibrium (LD) blocks, identification of enriched cell-types using ENCODE and Roadmap data and finally to assess functional pathways by performing gene set enrichment analysis.

The core idea is that by using SNPs as “seeds”, it is feasible to extract disease relevant processes by interrogating which genes, pathways, and epigenetic marks are localized in the vicinity of the established SNP´s. The rationale is that genetic variants, which have been identified in large cohort studies, provide us with one of the best and most reliable data-types with regard to diseases. Hence, by collecting molecular entities around those SNP´s we have the opportunity both to identify established pathways as well as finding novel candidate molecules and pathways or networks.

To this end, we collected published GWAS data from the US National Human Genome Research Institute (NHGRI) [45]. At the time of our analysis (18 January 2014) NHGRI contained 38 publications, which reported 171 SNPs above a significance threshold of *p* > 1 × 10^−6^. In addition, 1280 associations were considered for our analysis obtained from literature. Resulting in a total of 2282 distinct SNPs linked to 601 genes. These 601 genes were obtained by considering 20 kb region upstream and downstream of given variant, and constituted the starting list for further analysis. LD blocks were retrieved from HaploReg [46] with LD threshold, *r*^2^ = 0.8. Next, data for chromatin state dynamics have been determined using ChIP-Seq in a number of different cell lines as reported in ENCODE (ENCODE Project Consortium) and the Roadmap Epigenome Project [47]. This information can be used to link SNPs to epigenetic regulation in specific cell types. Cell specific chromatin states in six cell lines [48] were retrieved for AD or PD associated SNPs from ENCODE and Roadmap using HaploReg. Finally, pathway analysis of the SNPs data was performed using GSEA [49]. This dataset includes several gene products, which are involved in specific metabolic and signaling pathways. Using GSEA tools signaling or metabolic pathway were retrieved and then ranked based on BH corrected *p*-value. Not surprisingly, we observed that most of the AD associated SNPs (>73%) were found in intronic and intergenic regions of the genome, *i.e*., non-coding regions. For example, included in our analysis of the LD blocks we localized chr19: 45386467–45396144 representing the PVRL2 and TOMM40 gene region, which also had enrichment in the enhancer region suggesting regulation of brain tissue specific chromatin regulatory states in this zone. To assess the functional importance we analyzed the expression pattern of the implicated genes in CNS tissues. Interestingly, some the SNP-genes are expressed in liver cells. Sutcliffe *et al*. [50] have reported that the plaques that cause Alzheimer’s disease may originate in the liver, not the brain. In conclusion, a functional analysis suggests that the SNP associated to TOMM40 most likely interrupts the mitochondria-nucleus signaling pathway in AD. Finally, supporting this data-driven sequential finding there is an extensive literature supporting a role for mitochondrial dysfunction and oxidative damage in the pathogenesis of AD. For example, the review by Moreira *et al*. [51] discusses evidence indicating that mitochondrial dysfunction has an early role in Alzheimer’s disease. It has been shown that inactivation of TOMM40 evokes the mitochondrial unfolded protein response and causes a collapse of the proton gradient across the inner mitochondrial membrane [52].

#### 2.2.2. Multi-Partite Graph Mining: Heat Diffusion Approach

**Figure 1:**
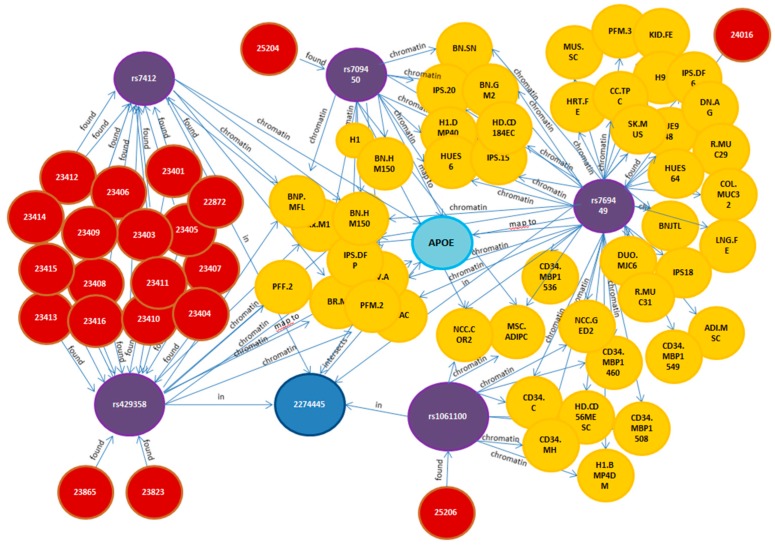
Head diffusion graph surrounding Apolipoprotein E (APOE). A graph representing Single Nucleotide Polymorphisms (SNPs) (purple circles), epigenetic information (chromatin states of genes), Linkage Disequilibrium (LD) blocks (blue circle), studies (orange circles) and cell types (yellow circles) displays relevant information associated with the APOE gene and its biology.

A sequential analysis scheme for data-integration, as presented in the previous section, holds the promise to identify novel mechanisms of disease beyond what is currently believed to be involved in AD. However, to scale such an approach in a systematic manner enabling an unbiased data-mining approach would be useful but challenging. To this end, we have developed what we refer to as a multi-partite-graph representation of several different data-types (Figure 1). Using such a data structure we benefit from techniques originating in statistical physics. In brief, the construction of analysis pipelines involving large volumes and multiple types of data frequently presents the problem of how to iteratively narrow the search field in order to select a smaller subset which can be analyzed in more detail. In heterogeneous data such selections are often made sequentially (as described in the previous section) using single data types, potentially discarding useful information. Hence it is desirable to find methods, which allow us to consider multiple data types and their associations concurrently.

Heat diffusion is an approach loosely based on classical thermodynamics for identifying “hot” nodes or sub-networks over data, which can be represented as a network. It is to be noted that, it has been used successfully in solving biological problems [53,54]. The data in question is converted into nodes (representing features such as genes, SNPs or functional annotations), and edges (representing associations between data nodes such as a SNP intersecting a gene). Edges can be assigned weights based both on the type of association they represent (a SNP to gene association might be considered inherently more important than an LD block to gene association), and specific properties of the association, for example a SNP to gene edge might be weighted differently depending on whether the SNP is exonic or intergenic.

To identify the nodes or sub-networks of interest, heat flow is simulated in the network. A prior distribution of heat is first chosen (such as SNPs already implicated in a disease). The heat simulation can be conceptually imagined with the nodes and edges represented as spheres and rods of some conductive material, with the prior nodes being heated. The heating is then removed and the network left until the heat distribution reaches a steady-state. Hot nodes or sub-networks can then be identified, and their significance tested by permuting the starting heat sources or edge weights.

It can be seen that it is still necessary to make informed but fundamentally arbitrary choices in constructing such a network representation, and choosing starting heat distributions, but that the choices made should be clearly documented and applied consistently. Such a method also necessarily discards much specific information about features and their associations (since all information has to be distilled to a single edge weight), but in return it provides a global view over multiple data types, which considers both the topology and properties of their associations, fairly identifying interesting subsets for conventional analysis. For AETIONOMY, we constructed a network consisting of AD or PD studies, SNPs, LD blocks, genes, Gene Ontology (GO) annotations, cell types and tissues. For each disease, SNPs previously implicated as having disease risk, and disease-associated GO terms and brain tissues were used as prior distributions, and the network used to identify “hot” genes. Using such a procedure we could recover AD genes as revealed from a sequential analysis (Section 2.2.1) as well as additional AD genes, which have been characterized by the AD community. Interestingly, the heat analysis readily provides novel candidates in the “vicinity” (based in the head diffusion) thus widening the scope of putative mechanisms of disease, which could subsequently be incorporated in modeling approaches, including those described in the following sections.

### 2.3. Model-Driven Identification of Disease Mechanisms

In 2011, Kola and Bell published a comment in Nature reviews drug discovery with the title “A call for a reform of the taxonomy of disease” [55]. In their communication, they question the current disease classification system, which is largely based on clinical phenotypes and that goes back to the work of William Farr in the middle of the 19th century [56]. Kola and Bell propose to replace the current disease classification system by mechanism-based taxonomies of disease, ultimately bearing the potential to resolve syndromes and to identify patient subgroups characterised by shared molecular aetiology. Such a mechanism-based taxonomy would effectively classify diseases according to the molecular and cellular aetiology of the disease, rather than using clinical features as the ordering principle (as it is currently the case with the ICD nomenclature [57]).

The concept is appealing in particular from the viewpoint of the pharmaceutical industry. The concept of a mechanism-based taxonomy goes far beyond the classical “target” paradigm that dominated drug discovery for the last 30 years. A classification system for human diseases based on pathophysiology mechanisms would provide the blueprint for better patient subgroup identification (through mechanism-specific biomarkers) and—most important—would deliver entire mechanisms that could be targeted by one or more compounds. It is therefore not surprising, that the pharmaceutical industry considered this concept attractive. In 2013, the Innovative Medicine Initiative (IMI) launched a call for proposals to generate mechanism-based taxonomies of disease and the AETIONOMY project is one of the winning proposals in this call. AETIONOMY aims at generating a mechanism-based taxonomy for two diseases (or better: syndromes): Alzheimer´s disease and Parkinson’s disease [58].

Of course, the challenge of generating a “mechanism-based taxonomy” for neurodegenerative diseases is enormous. Not only that neurodegenerative diseases are “idiopathic”, which means, that their aetiology is not clear but also the time course of the development of neurodegenerative diseases suggests that the first molecular dysregulation processes and the detectable clinical symptoms of the disease can be separated by more than a decade [59]. This fact and the fact that the amount of data from human samples is very limited and human biomaterial is usually derived from post-mortem brains, makes a data-driven approach to the identification of mechanisms of neurodegeneration almost impossible.

As outlined further above, identification of disease-mechanisms is likely to require multi-scale and multi-modal approaches. Moreover, we have learnt that there is not a single example of coherent data sets representing all biological levels, from the gene level via the protein level, the cellular physiology level, the tissue- and organ physiology level to the entire organism and groups of patients (population level). The absence of coherent data sets in the area of neurodegenerative diseases prompted us to look for alternative routes to identify putative disease mechanisms using integrative approaches. Neither purely knowledge-based nor purely data-driven approaches are likely to deliver the cause-and-effect relationships that we need for the identification of disease mechanism candidates.

In AETIONOMY, we therefore decided to combine knowledge and data to a model-driven approach [60].

#### 2.3.1. Multiscale Cause-and-Effect Modeling and Mechanism Candidate Identification

A key element of the model-driven approach towards identification of disease mechanisms in AD and PD is the use of OpenBEL, the open source version of the biological expression language [61]. OpenBEL is a language designed to capture, represent and analyze knowledge in a computable form, which means, that the formalism of knowledge representation supports causal reasoning algorithms [62]. OpenBEL focuses on causal and correlative relationships; use of the language is supported by a dedicated OpenBEL framework that provides essential analysis tools and algorithms [63]. One advantage of BEL over existing modeling languages is explicit support of semantics using domain-specific ontologies, which makes the encoding of biomedical features specific to neurodegeneration feasible by utilizing resources such as Alzheimer’s disease ontology [64] and Parkinson’s disease ontology [65].

OpenBEL is “scale-free” and can represent cause-and-effect relationships independent of levels and scales. As a consequence, OpenBEL allows for the integration and representation of knowledge derived from the molecular, cellular, organ and clinical level, which leads to higher resolution of information representation compared to similar modeling methods [66]. Moreover, OpenBEL is compliant with semantic standards, which makes it interoperable across multiple scientific disciplines. However, OpenBEL in its current form does not resolve processes over time nor does it distinguish between states of a system. Time-dependent processes (or comparisons between “normal” state and “diseased” state) can, however, be represented in two or more models that represent stages of disease.

Fundamentally, there are two different ways to use BEL-based cause-and-effect models for the identification of candidate mechanisms:

- Model-Model Comparisons

Model-model comparisons have been used by Kodamullil *et al*. [67] to identify candidate mechanisms possibly involved in the molecular aetiology of Alzheimer´s Disease. The approach is mainly based on an in-depth expert-inspection of the networks representing the “normal” and the “diseased” state of a neuron. This sort of model-model comparison bears the risk that it may be heavily biased towards the current research focus in the field. This research focus and the related publication intensity can be directly deduced from the graph density in the cause-and-effect models underlying this work (e.g., graph density around the amyloid cascade or the biology of the tau protein). The authors of that publication were obviously aware of the bias that comes with a knowledge-driven approach, but their analysis of the concordance (the relative fraction of observed gene expression regulation patterns compliant with the induction and repression relationships in subgraphs of the BEL models; for a detailed description of the concordance and richness network evaluation measures see Catlett *et al.* [68]) for publicly available gene expression data clearly demonstrated, that the publicly available gene expression data in the field of Alzheimer´s disease are non-informative when compared to the directional graph of the Alzheimer BEL model (Shweta Bagewadi, personal communication).

**Figure 2:**
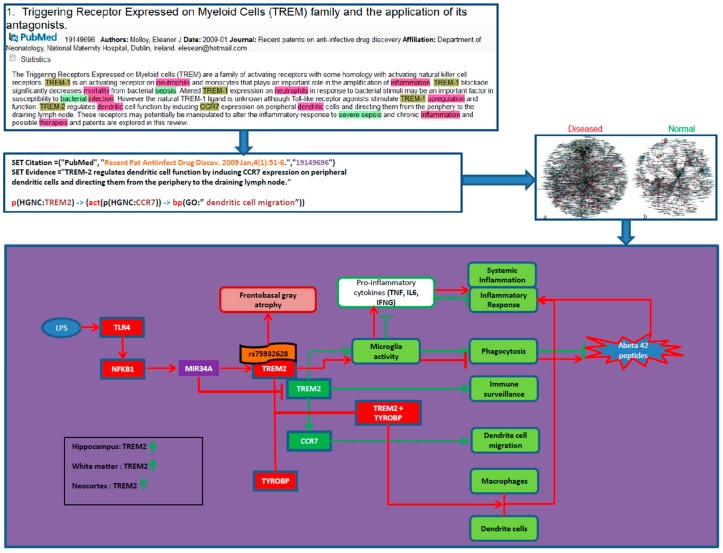
Differential analysis of triggering receptor expressed on myeloid cells 2 (TREM2) activities in neuroinflammation and normal state: In this figure dotted lines indicates regulation, arrows represent increase relation and T shaped lines represent inhibits/decrease relation. Red colored nodes and edges indicate the disease state mechanisms whereas green colored nodes and edges show normal state processes. The highlighted words in the uppermost screenshot represent the entity (term) markup in text by the text mining tool used.

Careful inspection of differences between the “normal neuron” and the “diseased neuron” models revealed “chains of causation” that represent putative candidate mechanisms. For each of these “candidate mechanisms”, additional evidences (e.g., from the patent literature, from mouse and cell-line model systems, from genetic analysis) were collected and the candidate mechanisms were ranked according to the number and strength of independent lines of functional evidence that support the notion of a role of that subgraph in the aetiology of disease. Figure 2 shows an example of a candidate mechanism identified by model-model comparison.

In normal state TREM2 regulates microglial activity and induces phagocytosis that removes the neuron debris like Abeta 42 peptides from brain. TREM2 regulates also inflammation by inhibiting proinflammatory cytokines such as TNF, IL6 and IFNG. In addition, it also maintains dendritic cell function by inducing CCR7 activities.

In disease state, LPS (lipopolysaccharide) induces TLR4, which increases NFKB1 activities. NFKB1 increases MIR34A which targets TREM2, decreasing normal TREM2 and increases the mutant variant. Recent GWAS studies associated SNP rs75932628 with TREM2 in LOAD patients. Also there are studies suggesting the link of this TREM2 variant with certain clinical and neuroimaging AD features such as frontobasal gray atrophy. Moreover, in disease brain TREM2 forms complex with TYROBP, which triggers immune responses through activating macrophages and dendritic cells which leads to chronic neuroinflammation.

- Reverse Causal Reasoning

Reverse causal reasoning (RCR) is an approach that aims at identifying upstream controllers of biological regulation patterns and other forms of “signatures” (e.g., functional context that has otherwise been described as a “pathway”). RCR has been developed to run data from high-throughput experiments (typically “omics-“ data) against a knowledge base represented by BEL networks.

The fundamentals of RCR have been described in great detail by Catlett and colleagues [68]; so that we refer to this essential publication for a detailed description of the algorithms underlying RCR. Here, we will only review some of the major features of RCR and point at some specific limitations for RCR-based candidate mechanism-identification in the area of neurodegenerative diseases.

RCR starts with a dissection of a large BEL network (representing some well-defined biology) into smaller subgraphs that could potentially be regulated by an upstream control node. In essence, the topology of the BEL graph is used for the generation of these subgraphs, which are called “hypotheses” [69]. Regulation patterns observed in omics-data, e.g., differential data sets that distinguish between a “normal” and a “diseased” state, are being mapped to these hypotheses. The results of these mappings are evaluated and hypotheses are ranked according to their ability to explain the data. As BEL is simplifying the world of biology (by discretizing regulation events into 5 classes: “up-regulation”, “down-regulation”, “direct up-regulation”, direct down-regulation” and “no regulation”), the mapping of data onto the knowledge-graph representing the hypothesis allows for a classification of the following types of discrete compliance states between data and knowledge: “increase” (+), “decrease” (−) and “ambiguous” (?).

This abstraction of complex regulation processes allows for a comparably straightforward scoring of the compliance of a knowledge-encoded regulation state and the regulation state that is found in the data. It is clear that such regulation events are predominantly available in gene expression (mRNA expression) data, but other data types (e.g., proteomics experiments) that represent states and state changes can be used for that procedure, too.

Two evaluation indicators provide a means of the strength and consistency of the support that the mapping of the data to the hypotheses results in, or, in other words: the evaluation indicators are quality measures for the model (hypotheses) validation through the available data.

“Concordance” is a statistics value (calculated as a *p*-value) to determine the consistency of the observed states (from the differential data) of the downstream nodes with the direction assigned to the hypotheses upstream (controller) node. The other indicator is “richness”, which describes the relative proportion of concordant state changes in a hypotheses compared to all state changes in the population of mRNAs assayed in the gene expression experiment. “Richness” therefore indicates, how “important” or how “prominent” an observed regulation pattern is given the mass of all state changes (e.g., indicated by differential gene expression) in the high-throughput data set.

BEL has been successfully applied for the identification of upstream control elements in hepatocytes [70], for the identification of biomarker candidates [71], for the mechanistic interpretation of drug responses [72] and for patient stratification [73]. All these examples have in common that they have been generated in information—rich domains (oncology, immunology); the application of OpenBEL in neurology, however, is even more challenging, as the number of publicly available, informative omics-experiments is very limited.

For the integration of genetic variation information, we have recently modified the OpenBEL syntax [67]. Reasoning over genetic variation information in a network biology approach is a non-trivial task; we therefore focused on the integration and representation of SNPs in BEL models; effectively supporting a human intelligence based interpretation of cause-and-effect relationships that take SNPs into account. Future efforts in our group will aim at development of algorithms that allow for direct causal reasoning over genetic variation; however, biological impact assessment of SNPs that can be mapped to objects in BEL models remains a major challenge in particular for intergenic SNPs [41].

The integration of genetic variation information in BEL-based “cause-and-effect” models has been used by our group to systematically identify “shared mechanisms” that are candidate mechanisms for co-morbidity phenotypes. SNPs that were associated with both, AD and type-2-diabetes mellitus (T2DM) were systematically identified and mapped to a comprehensive model of Alzheimer´s disease [67]. Through modeling of major T2DM pathways, BEL subgraphs could be identified that overlap between AD and T2DM and that share SNPs (or LD blocks) that can be associated with genes and proteins contained in the BEL models.

Graph-Mining for the Identification of Important Markers in Context: NeuroRDF.

Rapid advancements in high-throughput technologies for quantifying biomolecules have fuelled a dramatic accumulation of large volumes of heterogeneous data types. To leverage such data to help unravel complex disease mechanisms necessitates appropriate standards for data integration and exchange. Semantic web technologies such as Resource Description Framework (RDF) [74] have been employed to publish and link heterogeneous data [75]. RDF follows a simple triple format for representing relationships between entities: subject, predicate, object. The entities themselves and their relationships are encoded using Uniform Resource Identifiers (URIs) which are provided by domain-specific ontologies. Recently established resources such as Identifiers.org provide resolvable and persistent URIs to the community. Sharing such uniquely resolvable URIs enables different domain RDF graphs to be linked and traversed using query languages such as SPARQL. In response to these developments, a series of BioHackathon workshops [76,77] were organized to bring together key life science resource providers such as the EBI [78] and OBO Foundry [79] to publish their data as RDF. Furthermore, the Open PHACTS consortium [80] emerged in 2011 to provide an open-source, RDF-based drug-discovery platform to integrate pharmacological data. The successful outcome of these initiatives and hackathons advocate the utility of RDF as a scalable infrastructure, capable of integrating a wide spectrum of heterogeneous (semi-) structured and unstructured data. Extension of domain ontologies is sometimes needed to fill the gaps between existing resources, especially as new data modalities arise, to improve linking for subsequent analysis.

Harnessing the potential of RDF, Iyappan *et al.* [81] have recently developed an integrated semantic model encompassing multiple, complementary molecular data types for different neurodegenerative diseases (*NeuroRDF* [81]). These data include literature-derived Protein-Protein-Interaction (PPIs) networks, MicroRNA-Target-Interactions (MTIs) networks, transcriptomic data from GEO [82] and ArrayExpress [83], and knowledge from established biological databases such as MINT [84], IntAct [85], *etc.* Querying such an integrated resource can support efforts to elucidate new disease mechanisms. However, the quality of the derived mechanisms depends strongly on precise, highly curated meta-information for the integrated data. Therefore, the authors expended considerable effort to review each data source reported in the Alzheimer’s disease use-case. Overall, the authors spent in total 2 years of effort to retain high quality data.

The advent of DNA Microarrays (e.g., Affymetrix GeneChip) around the turn of the century made it possible to survey, for the first time, gene expression in a systematic, comprehensive and efficient manner. For well over a decade now, such platforms have been the mainstay of research into systems-level transcriptional changes in a huge variety of biological contexts. Although Next Generation Sequencing alternatives are increasingly succeeding the use of spotted arrays, such platforms remain popular and a considerable combined legacy of transcriptomic data is readily available to the research community (GEO, ArrayExpress [83]). The combined analysis of multiple, unbiased quantifications of the transcriptome from diseased patients, provides a valuable complement to knowledge-driven approaches, to generate novel insight into disease patho-mechanisms. However, meta-analysis approaches rely on the precise selection of a set of studies with a biological justification for their combined analysis. Zheng *et al*. [86], in a 3-stage subtractive approach, consider gene expression data from laser captured dopaminergic neurons of severe Parkinson’s disease patients and subsequently similar samples from incipient disease and other brain regions that display Lewy body pathology, in order to implicate PGC-1A as a potential therapeutic target for early intervention. To faithfully identify the correct studies to include in such a meta-analysis strategy requires detailed, domain-specific meta-data for each study, which is only partially addressed by the MIAME standard. To enrich the available meta-data for Alzheimer’s disease gene expression studies, Bagewadi *et al.* [87] followed a semi-automated, manually curated approach to harvest detailed and standardised annotations from the accompanying publications. The annotations are modeled using a custom RDF schema that allows in-depth assessment of the respective phenotype, the type of tissue used in an experiment, and accessory information for the donor of the tissue such as; gender, age, possible comorbidities, *etc.* in addition to derived statistical measures such as fold change and adjusted *p*-values.

A disease mechanism is the orchestrated action of a collection of interacting biological molecules. Systematic mapping of protein interactions in different functional contexts can serve as a priori knowledge framework for the interpretation of quantitative molecular datasets such as gene expression data. A considerable resource of protein interactions involved in different biological processes can be found as unstructured text in published articles. Although, text-mining methodologies allow sensitive retrieval of different biological entities from published articles, capturing the context in which those entities are described remains inaccurate due to technological limitations. For *NeuroRDF* automated text mining was used to identify PPIs [88] and MTIs [89] in AD articles before manual curation to confirm the disease relevance and capture associated meta-information. The meta-information such as species, experimental technique used, tissue specificity *etc.* support in investigating interactions with more granularity. Disease PPIs reflect perturbations of the molecular networks underlying normal physiology. They may highlight key regulators but are an incomplete picture of the affected mechanism which normal state interactions matched to anatomical regions can help fill-in. Therefore, again the authors integrated a PPI network representing the healthy state (derived from 21 databases) in *NeuroRDF*. Such integration should always be done in a very cautious way as database content often suffers from a lack of specificity and accuracy. For instance, Younesi *et al.* [90] invested considerable efforts to retain highly qualified PPIs specific to the healthy human brain, which were used for correlating image-based diagnoses to their corresponding PPIs specific to brain regions.

**Figure 3:**
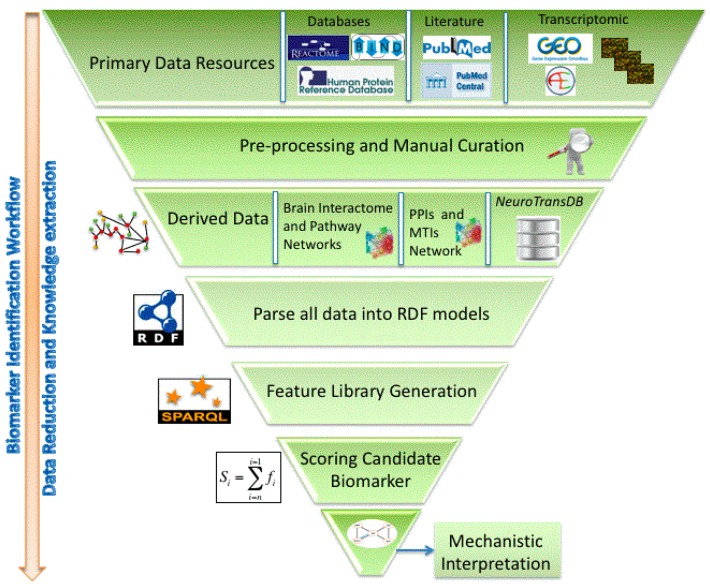
Workflow for Resource Description Framework (RDF)-mediated data and knowledge integration and the identification of “signals” in a network context. The workflow essentially identifies subgraphs in the feature network that are highly discriminating between healthy and diseased; the term “long list” describes a list of “interesting network features” that provide contextual, mechanistic insight that can be analyzed by experts in the field.

A RDF model that integrates data for complementary molecular entities, together with carefully curated and enriched meta-data can aid research into disease patho-mechanisms. The RDF semantic query language, SPARQL, can be used to formulate common considerations that relate to a molecules biological role in disease, as queries and evaluate them systematically. The extracted features can used to objectively prioritise existing mechanistic hypotheses as well as uncover more novel etiologies. Such an approach has been employed by the authors as part of the Neuroallianz [91] consortium to extract a comprehensive set of gene features by querying *NeuroRDF*; incorporating annotations from *NeuroTransDB*, in order to prioritise gene products as potential biomarkers in AD. Extracted features included basic meta-analysis metrics such as median fold change; knowledge-based features such as targets of miRNAs dysregulated in AD; and metrics from the disease PPI network such as node degree and betweenness centrality. A rationally informed weighting scheme was used to combine the different features to produce a final score to prioritise each gene as a putative AD biomarker (manuscript in preparation) (Figure 3). It is also possible to express more complex biological questions as SPARQL queries across different entity classes in the *NeuroRDF* data, in order to elaborate existing disease mechanisms. The authors have tried to emphasize such complex multi-entity relationship querying using amyloid-beta hypothesis use-case in Alzheimer’s disease. In order to elaborate the APP mechanism at systems level, queries were issued to filter for genes that were differentially expressed, regulated by miRNAs, depicting strong topological properties such as high degree, *etc.* This enabled them to prioritize SHC1 gene, which has been extensively studied in cancer [92] and insulin resistance [93], but very limited in context of AD. Denoting SHCI’s involvement in shared-mechanisms around the commonly reported players: APP, BACE1, PRNP and LRP1 (derived from AlzGene [94] database), makes it a novel candidate for further investigations.

#### 2.3.2. Mechanism-Identification through Data Overlays: The PD Map Approach

Parkinson’s disease (PD) is an idiopathic disease where individual interplays of different cellular processes and molecular pathways combine into the multi-factorial pathogenesis of the disease. Thus, it is not possible to identify disease-related perturbations in cellular processes by just analyzing single entities such as protein abundance or gene expression, as done in the classical biomarker approach. Large-scale data could help to identify perturbations of disease-specific cellular pathways and processes by integrating data from numerous experiments and publications into detailed disease-related molecular networks, in reference to the mechanism-based taxonomies of disease as outlined above in Section 2.3. The challenge is to identify the causative perturbations in this complex picture, be it in signal transduction pathways or cellular processes. Different tools enable the identification of perturbed pathways and processes from large-scale datasets, however, their output is often limited to ranked lists of associated pathways (e.g., based on GO annotations) without providing disease-related information or showing potential cross reactivity between pathways. In addition, the impact of differential gene expression and protein activity may vary substantially dependent on a specific tissue, cell type or a subcellular compartment. This fact is often neglected.

To avoid this potential pitfall, data analysis and visualization should allow for a more specific and targeted approach, with regard to disease-related mechanisms and networks. To fulfill this need, the PD map, a manually curated, fully annotated knowledge repository, was established [95]. The map describes detailed molecular mechanisms involved in the pathogenesis of PD. The PD map is based on the CellDesigner software [96], which is compliant to SBML (Systems Biology Markup Language) and SBGN (Systems Biology Graphical Notation) [97]. Currently it contains more than thousand PD-related molecular interactions curated from over a thousand PD-related publications. The PD map displays molecular interactions within a cellular and subcellular context with a focus on cellular processes implicated in PD pathogenesis such as synaptic and mitochondrial dysfunction, impaired protein degradation, α-synuclein pathobiology and neuro-inflammation. It is the first freely accessible and high quality knowledge repository of molecular mechanisms contributing to the complex pathogenesis of PD [98].

Large-scale datasets derived from different experimental approaches such as proteomics, transcriptomics or genomics (e.g., GWAS analysis) can easily be uploaded to the PD map by any user. The differential expression is displayed on the PD map in the context of the cellular pathways and processes that are modeled in the map. The map provides disease and cellular context-related information in such a way that it is useful for data interpretation and hypothesis generation.

**Figure 4:**
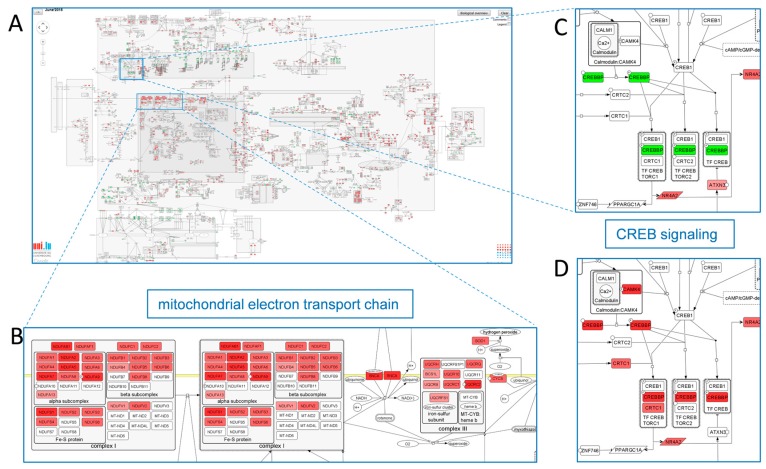
Data upload to Parkinson´s disease (PD) map (June 2015 release). (**A**) Differential gene expression (false-discovery-rate (FDR) <0.05) from a meta-analysis of eight transcriptome data sets, comparing human post-mortem brain samples from PD *vs.* healthy controls displayed on the full PD map [95]; (**B**) Section of the mitochondrial electron transport chain; (**C**) Section of the cAMP responsive element binding protein (CREB) signaling pathway; (**D**) Age-dependent differentially expressed genes from healthy aging brain [98]. Section of the CREB signaling pathway. Color-code: green = up regulation, red = down regulation.

An example for the use of the map is given by a meta-analysis of eight genes expression data-sets from post-mortem brain tissue and the mapping of the resulting differentially expressed genes onto the PD map. Different molecular processes such as autophagy, ubiquitin proteasome system and dopamine metabolism could be identified in the PD map to be affected in the diseased tissue. Most pronounced, a clear perturbation of mitochondrial respiratory chain is detectable pointing to mitochondrial dysfunction in the diseased brain; one of the hallmarks in PD (see Figure 4A,B). Within a further application the disease-related expression were compared to age-related effects. Differences in distinct pathways such as the CREB (cAMP responsive element binding protein) pathway, involved in neuron maintenance and mitochondrial biogenesis could be identified [98]. In older people induction of CREB pathway is down regulated whereas in PD this pathway is induced but without having an impact on the downstream NR4A2 pathways (Figure 4C,D). Based on this analysis the CREB signaling could be selected as a potential target for further investigations on the PD aetiology. One hypothesis that could be deduced from the PD map points to a potential involvement of the CREB inhibitor Ataxin-3 which is described to be involved in the neurodegenerative Machado-Joseph disease. Interestingly, Ataxin-3 is down regulated in brain tissue from PD patients. Both datasets derived from post-mortem brain tissue and aging are integrated in the PD map (layouts view) and can be explored by the PD map user.

Due to the embedded functionalities, the possible applications of the PD map are diverse. Identification of specific pathway perturbation based on data derived from patients could support the stratification of patients. Additional knowledge, like drug targeting information is useful in particular within pharmacological investigations. The PD map can also be used to visualize and contextualize results that are deduced from metabolomics experiments or from related databases such as RECON2 [99]. To ensure the functionality of the PD map the database needs to be updated on a regular basis. Text mining approaches in combination with expert curation are essential to digest the extensive literature and integrate valuable information into the PD map. Finally, the PD map framework is already used as a blueprint for other diseases maps.

#### 2.3.3. The Pathophysiology Graph: Representation of Multiscale Physiology Using ApiNATOMY

**Figure 5:**
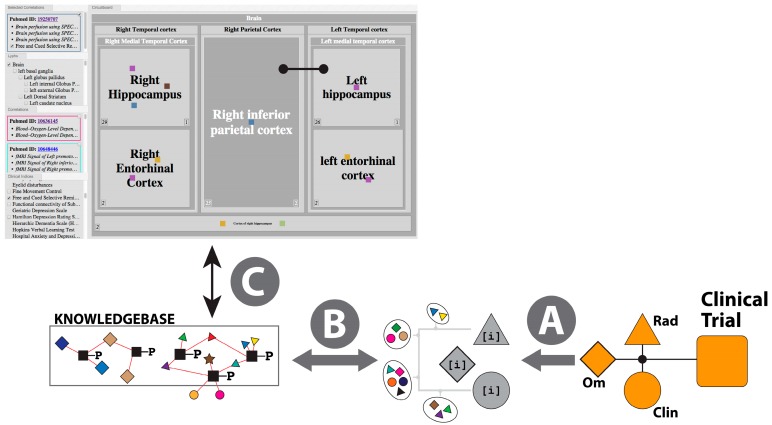
Workflow for the interpretation (Step **B**) of results from clustering analysis of NDD patient data (Step **A**); through the application of physiology route calculations and visualization in ApiNATOMY (Step **C**). bottom right: Triangle represent radiological measurements; Circle represent clinical indices, Diamond represent “omic” indices. The orange color is meant to describe the generic character of the abovementioned dimensions. In the other parts of the graphic, the various colors in the shapes are representing the values that are taken by those dimensions in a specific context (e.g., individuals). The P is an abbreviation for Publication.

A key function of the pathophysiology graph is to provide a verifiable, computer-readable knowledge scaffold across multiple scales of structures and processes in which these structures participate. In effect, this scaffold contributes critically to the mechanistic interpretation of patient subgroups that are identified through clustering of linked clinical, imaging and molecular indices collected in clinical studies (Figure 5). The core requirement of this interpretative step is to generate hypotheses of multiscale mechanisms that explain why, for instance, PD-type pathology is manifested differently in distinct patient sub-groups.

The development of a pathophysiology graph that provides for the above requirements has to address a number of challenges, namely to:

(a) Map multi-modal measurements onto the same formal representation of brain multiscale location (*i.e*., anatomy)—in Neuro-Degenerative Diseases (NDD) the primary focus is on the anatomical integration of “Omics“-type measurements (e.g., gene expression) and radiology measurements (e.g., thicknesses of neocortical regions);
(b) Organize knowledge about multiscale routes of interaction between different brain locations to objectively compare and contrast physiological and pathophysiological processes—in NDD, knowledge of such routes is critical to track flows of (i) fluid, at the basis of molecular interaction and pathological agent spread; as well as (ii) electrical spread, as the basis of neuropsychometric tests applied to monitor symptoms and signs of disease progress;
(c) Coherently bridge neuropsychometric tests to the underlying neural substrate responsible for behavior observed in NDD patients—given that a specific pathology underlying NDD may express different spread patterns in different patient subgroups, neuropsychometric scores (e.g., memory recall tests) provide an important means to track such spread;
(d) Take into account the lack of functional symmetry of brain anatomy, with particular reference to the asymmetry that typifies the involvement of brain structures in behavior and pathology.

Given the above challenges, the ApiNATOMY toolkit [100] is being applied to provide the requiste knowledge management infrastructure and services for hypothesis generation for multiscale mechanisms in NDD, but supporting knowledge curation, inferencing and visualization [101]. These three areas of contribution are discussed below:

(i) Knowledge Curation: Clinical Scores

This area is focused on the application of ApiNATOMY tools to contribute clinical knowledge to the AETIONOMY knowledgebase. ApiNATOMY-based editors support the collection and collation of literature-derived knowledge from publications that correlate clinical scores with multi-modal measurements from the brain. This curational component has two key steps, namely to: (1) create OWL-based classes that link qualities being measured with the location from where the measurement originates; and (2) instantiate data objects that link lists of these brain -located measurement with clinical scores. A core curational principle is to always capture brain region lateralization to ensure that any asymmetry in the brain’s involvement in behavioral manifestations is recorded.

(ii) Inferencing for Hypothesis Generation

The above curational effort gives rise to a network of correlations between clinical scores and multi-modal measurements from the brain, such that the same brain region may be involved in multiple clinical correlations. The role of this knowledgebase in AETIONOMY is to provide mechanistic hypotheses that explain the recurrence of combinations of values of particular clinical scores and/or brain-located measurements that typify specific NDD patient subgroups (e.g., as identified by clustering of clinical study data from initiatives such as ADNI [102] or PPMI [103]). By importing brain tract data, from sources such as the Neuroscience Information Framework, the mechanisms proposed by ApiNATOMY will consist of those networks of tract-connected brain regions that most parsimoniously explain the above combinations of values identified by clustering of study data. The procedure to generate scored mechanistic hypotheses covers the following components:

(a) A lateralized topological model, generated from the combination of human, monkey and rat connectivity data, is built to provide a weighted reference graph that represents brain regions (about 400 regions) and their pairwise connections (about 4000 connections);
(b) The weighted graph in (a) provides the reference map for the application of a Steiner Minimal Tree (SMT) calculation [104,105] that finds parsimonious solutions of paths that link an arbitrary set of brain regions, such that preference is given to connectivity edges in this order: human, monkey, then rat. Given that the SMT problem is known to be NP-complete, we allow for the application of heuristics in the context of NDD calculations [106,107,108], where deemed appropriate—for instance, these heuristics also take into account expert knowledge about the likely direction of spread of NDD pathology from central, lower nuclei towards higher neocortical grey matter;
(c) For each clinical correlation data point, the most parsimonious route (or set of top-scoring routes) linking the brain regions associated with the brain-derived measurements that correlate with the clinical scores is calculated as described in (b) above, to create the correlation reference route set (CRRS);
(d) The calculation of a mechanistic hypothesis for NDD, then, takes as an input combinations of clinical scores and brain-derived measurements that have been clustered from clinical study datasets. The likelihood of mechanistic relevance of the route connecting brain regions (i) correlating with clinical scores; and (ii) from which measurements originate, is scored on the basis of the quantifiably-improved parsimony of (a) the *de novo* SMT solution for these brain regions compared to (b) the non-redundant graph merger of the independently-derived clinical-score-specific routes from the CRRS.

(iii) Visualization

There are a number of challenges in providing the graphical user interfaces (GUIs) in support of the navigation of multiscale, multi-modal data. To provide an anatomically-meaningful map that conveys functional information with visual immediacy, the ApiNATOMY toolkit makes use of a treemap-based interface that navigates nested tiles representing the mereotopology of lateralized brain regions. This approach is specifically tailored for the depiction of multiscale anatomy, overcoming the expressive limitations of generic software such as Cytoscape [109] or Spotfire [110]. In the ApiNATOMY interactive GUI, these tiles hold (i) graphical symbols for different kinds of multi-modal measurements (e.g., those measurements that correlate to neuropsychometric scores) taken from the brain regions that the tiles represent; as well as (ii) graphical edges representing either molecular networks derived from BEL representation of regionally-relevant molecular processes, or neural tracts that link one region with another (graphical depiction of a multi-site neural tract depicted in Figure 5).

#### 2.3.4. The AETIONOMY Knowledge Base—An Environment for Candidate Mechanism Identification

In the context of IMI project AETIONOMY [60] we have assembled a complex workflow for candidate mechanism identification that comprises some of the above described approaches. The knowledge base consists of a tranSMART component [111] (contributed and enabled through the eTRIKS [112] project) and additional functionalities that support model-driven (contextual) analysis of data and that makes data and knowledge completely interoperable.

The AETIONOMY Knowledge Base contains highly curated data [73]. Curated data represent molecular (“omics”) entities and their interaction and clinical data (including trial data). A comprehensive, semantic framework representing and formalising knowledge about anatomy (FMA [113], BRCO [114], NIF [115]), about major neurodegenerative diseases (ADO [64], PDON [65], MSO [116]) and about assays and readouts (variables) used in clinical trials (NDD-CTO [117]; NIFT [118]; clinicaltrials.gov [119]) enables “shared semantics” over all scales (from the molecular via the cellular and organ level to the cohort and population level) and all entity types (ranging from genes and proteins to cognitive testing and neuro-imaging).

A “model store” harbors various disease models available to the project (AD and PD models in OpenBEL; a PD map in SBML). The model store for BEL models is based on a relational database schema, which makes this type of disease models amenable for SQL-based querying and enables an unprecedented integration of models and databases.

A growing number of relevant public databases (e.g., CTD [120], UniProt [121], WikiPathways [122], DrugBank [123], HGNC [124], PubMed [125], Reactome [126], proteinAtlas [127], NCBI taxonomy [128], ChEBI [129], InterPro [130], various PPI databases [131,132]) have been imported in this system; extensive mappings between these databases allows for seamless integration of models, referential databases, and data analysis results coming from the tranSMART component.

**Figure 6:**
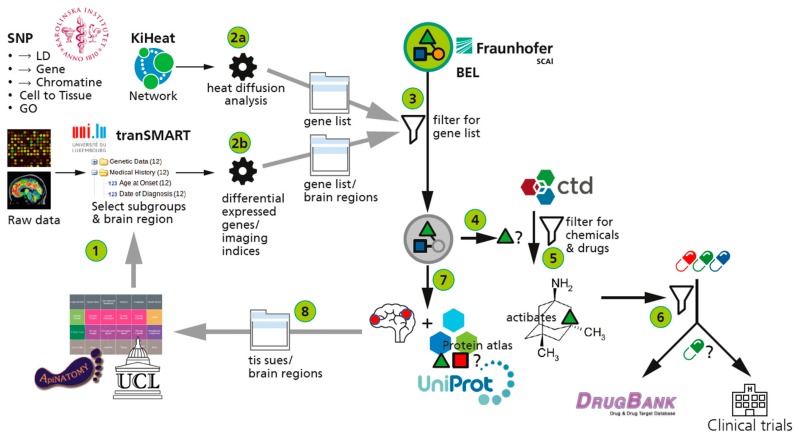
Workflow for candidate mechanism identification in the AETIONOMY Knowledge Base—the Parkinson Disease example. 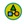 represent BEL ans SBML models; 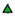 represents the gene component of the model. 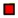 represents the protein component.

Figure 6 shows the overall workflow that is supported by the AETIONOMY KB: starting with candidate gene lists that result from, e.g., differential gene expression analysis performed within the tranSMART component or that can result from the SNP mining approach described above, the disease models are queried for the representation of functional context around these candidate genes (or other genes of interest, e.g., biomarker candidates). Mapping of gene and SNP identifier to the OpenBEL models containing gene and SNP information allows for the identification of BEL subgraphs that represent functional context around these genes of interest. Automated path search algorithms expand the network around those candidate genes that can be mapped to the models; the resulting subgraph can also be enriched by brain-region specific information. OpenBEL subgraphs represent functional context which is not constrained to molecular processes, but also comprises often anatomical and cell-type specific information, which can be mapped to the physiology graph underlying the ApiNATOMY approach. Starting the workflow with either SNPs or differential gene expression analysis is only one way to use the information integrated in the AETIONOMY KB; other “seed entity lists” could stem, e.g., from pattern analysis in clinical data or neuro-imaging data.

SNP data analysis and differential gene expression analysis done in the tranSMART component of the AETIONOMY KB lead to candidate gene lists (2a and 2b), that comprise already anatomical location information in some instances. The differential gene expression lists and the SNP analysis lists contain identifiers, that can be readily mapped to identifiers underlying objects in disease models. Disease models encoded in OpenBEL are being queried using the candidate gene lists (3). Subgraphs representing functional context and containing at least one of the genes identified in differential gene expression and SNP association studies are being identified. Significant SNP loci or interesting differentially expressed genes are immediately “embedded” in a functional context using this approach. Subgraphs are either linked to the toxicogenomics databases CTD (4) or mapped to UniProt protein atlas information (7). The mapping to brain-region-specific PPIs allows us to put functional (molecular) context into an anatomical site; with high likeliness of brain-region specificity of the interaction predicted by the BEL model. The CTD mapping allows to identify targets and drugs that can be associated with the functional context in that subgraph; for drug target combinations linkouts to Clinical Trials and DrugBank provide useful information on their indication and efficacy. Note that the entire workflow does work for Alzheimer´s Disease in similar ways.

The internal mapping of entities represented in the subgraph to other databases allows for an identification of targets using the link to CTD. Whether these targets have already been tested in the context of neurodegenerative disease trials is being analyzed through a mapping and querying of ClinicalTrials.gov [119].

The AETIONOMY Knowledge Base integrates multiscale data and knowledge to an extend that complex mining tasks are enabled; future developments will also integrate imaging—derived features in both, BEL-based models and on the data side (by incorporation of major clinical study data such as ADNI and PPMI into the knowledge base; access to these resources will, however, be restricted in order to comply with the patient data privacy regulations that have been associated with the scientific use of these data sets). Additional clinical trial data will come from the pharmaceutical industry partners in AETIONOMY; together, this will form an unprecedented example for integrative data and knowledge management in the area of neurodegenerative diseases.

At this point, we would like to re-emphasize on the need to critically review and to curate the data before they are entered into a knowledge base such as the AETIONOMY KB. We have spent more than three years of time and a labor power of more than 10 man years on curating all publicly available omics data that can be linked to the human, the murine and the rat brain. Of course, we will further improve annotation and curation guidelines and make those publicly available; most of the content of the AETIONOMY KB is freely available for research purposes anyway.

## 3. Conclusions

The ambitious mission that we have assigned to ourselves is to tackle the deciphering of neurological disease mechanisms. The difficulty in this field is mainly due to the availability of data which is scarce. On the one hand due to difficulties to access samples in relevant conditions: e.g., the inherent impossibility to access brain tissues from patients in the course of the disease development, and on the other hand there is a lack of data from individuals who have been followed over time while different data-modalities have been sampled from the same individual. This situation contrasts in comparison with the field of oncology for example, where there is a huge wealth of accessible individualized data of several types.

As we are mentioning the oncology field and as we are using a wide variety of technologies, it is interesting to mention that some of them are borrowed from other fields: e.g., heat diffusion was utilized for sample classification of TCGA data. In addition, with the uprising of the immune-oncology discipline, our colleagues from oncology are currently revisiting the huge amount of data that they own. Their goal is to analyze the tumor infiltration by immune cells in order to perform a “gene/infiltrating immune cell type” signature based stratification of the tumors. Galon *et al*. [133] have performed such analysis and they have also added the temporal dimension to the spatial dimension in the context of colorectal cancer. The final goal is to maximize chances of survival of the patients by performing a more precise analysis of the tumor environment in complement to the TNM classification, which shall lead to more adapted treatments according to the tumor context and its temporal changes. This work could be extended by using some of the techniques that we are currently applying and further developing: openBEL-based multiscale cause-and-effect modeling and the pathophysiology graph concept would certainly be of value and bring a mechanistic interpretation layer to the development of tumors and allow to unravel the interplay, which exist between tumor development and the immune contexture.

As illustrated in this review, a strategy to deal with this situation for neurodegenerative diseases, we are therefore not following a single methodology to integrate and interpret the wide variety of data that we are taking in account but we are using a multiplicity of methodologies from which we get a quite wide range of results. There is sometimes confluence between the various methods, which may be interpreted as increasing the robustness to a given hypothesis but this does not prevent us from having valuable results, which are specific to a method. In that case, the hypothesis may be due to the higher sensitivity of given a method in a specific context and the result may be considered as relevant too: it is the accumulation of secondary evidence which will enable the decision to preserve or reject the hypothesis.

By definition, we have chosen to integrate if not all dimensions, at least a large amount of the available information coming from many modalities and many scales (CSF biomarkers such as TAU, pTAU, Aβ42, image indices like brain subregion volumes, LD scores for SNP, results from differential expression analysis, *etc.*). We are so forth obliged to work with multi-scale and multi-modal indices which are increasing the complexity of the task but we are developing agile strategies which enable us to reconfigure our processes in a timely manner. We can use the algorithms that we have described above as a chain: each result of one step being the input for the next one: in that case each steps acts as a filter and we are restricting at each steps the number of possible results to focus to the most relevant one. We can also use them as independent steps from which results have to be collided. It is to be noticed that the second option can be realized by integrating many dimensions in one step when using the “heat-diffusion” algorithm by taking a composite heat vector: this corresponds to a holistic analysis of the modalities and scales.

In addition, we are currently creating a database of mechanisms underlying neurological diseases, while having a particular emphasis on AD and PD, our approach can be extended to other neurological diseases. This database (which is yet unpublished) is composed of around two hundred BEL subgraphs which represent relevant mechanisms in given contexts on top of which we want to add all the relevant documentation. In the near future, we obviously want to test the subgraphs—hypotheses for diseases—using real life data and we will use each mechanism of our database as a probe which can be challenged using the available datasets (e.g., ADNI, PPMI) or clinical studies made available to the AETIONOMY project.

Finally, the “classical medicine” has already brought a huge wealth of drugs to the living community. The approach advocated here can be considered as a way of thinking and is shared by others, e.g., our PRECISEADS colleagues, fitting with the general ambition of fully entering the precision or personalized neurology.
